# Pulmonary artery involvement in Takayasu’s arteritis: diagnosis before pulmonary hypertension

**DOI:** 10.1186/s12890-019-0983-7

**Published:** 2019-11-27

**Authors:** Jing Yang, Min Peng, Juhong Shi, Wenjie Zheng, Xuezhong Yu

**Affiliations:** 1 Department of Emergency, Peking Union Medical College Hospital, Chinese Academy of Medical Sciences and Peking Union Medical College, No. 1 Shuaifuyuan, Dongcheng District, Beijing, 100730 China; 2 Department of Respiratory and Critical Care Medicine, Peking Union Medical College Hospital, Chinese Academy of Medical Sciences and Peking Union Medical College, No. 1 Shuaifuyuan, Dongcheng District, Beijing, 100730 China; 3 Department of Rheumatology, Peking Union Medical College Hospital, Chinese Academy of Medical Sciences and Peking Union Medical College, No. 1 Shuaifuyuan, Dongcheng District, Beijing, 100730 China

**Keywords:** Pulmonary arteritis, Pulmonary hypertension, Takayasu’s arteritis, Chest CT scan

## Abstract

**Background:**

This study was performed to analyze the clinical manifestations, imaging features, and prognosis of Takayasu’s arteritis (TA) with pulmonary arteritis (PA).

**Methods:**

In total, 51 of 815 patients with TA were diagnosed with PA at the Peking Union Medical College Hospital from 1986 to 2015. The patients’ medical records and radiographic data were retrospectively reviewed.

**Results:**

The patients comprised 39 women and 12 men with a median age of 33 years (range, 14–67 years). The most common symptoms were dyspnea (70.6%), cough (66.7%), hemoptysis (47.1%), and chest pain (45.1%). Computed tomography (CT) pulmonary angiography, pulmonary arteriography, and pulmonary perfusion imaging showed pulmonary artery stenosis or occlusion in 44 patients. A total of 82.4% of patients had lung parenchyma lesions on CT scans, indirectly indicating pulmonary artery involvement. Additionally, 58.8% of patients had pulmonary hypertension (PH) by echocardiography. Compared with the PH group, the non-PH group was characterized by a shorter disease duration; more symptoms such as fever, chest pain, and hemoptysis; an increased erythrocyte sedimentation rate; and a higher incidence of subpleural wedge-shaped shadows on chest CT (*P* < 0.05). The median follow-up period was 48 months (range, 1–212 months), and all three deaths occurred in the PH group.

**Conclusions:**

The clinical manifestations of TA with PA are nonspecific. PH often complicates PA and is associated with a poor prognosis. Early clinical manifestations such as repeated fever, chest pain, hemoptysis, and recurrence of subpleural wedge-shaped shadows on chest CT should arouse suspicion of PA in patients with TA and prompt further investigations. This may allow PA to be diagnosed before the occurrence of PH.

**Trial registration:**

*ClinicalTrials*, NCT03189602. Date of registration: June 16, 2017. Retrospectively registered.

## Background

Takayasu’s arteritis (TA) is a form of chronic vasculitis primarily involving the large arteries and their main branches, and the pathological manifestation is arterial full-thickness inflammation [1]. TA can affect the aorta, subclavian artery, renal artery, iliac artery, coronary artery, and other blood vessels. The clinical manifestations vary greatly depending on the area, severity, and duration of vascular involvement. TA usually progresses through three stages [2]: an early stage characterized by nonspecific symptoms, a vasculitis stage characterized by a systemic inflammatory response and thickening of the vascular walls, and a static stage characterized by dissipating inflammation and occlusion of affected vessels. Notably, the vasculitis stage is a crucial period for diagnosis and treatment.

TA can involve the pulmonary artery [3, 4]. The incidence of pulmonary arteritis (PA) in patients with TA varies greatly among studies (0–56%) [5, 6]. Pulmonary hypertension (PH) occurs in 12 to 13% of patients with TA and in 42.2% of patients with PA [3, 6, 7]. No large-sample study of PA and PH in patients with TA has been performed to date. Only one Chinese hospital specializing in cardiovascular diseases reported that the incidence of PH in patients with TA involving the pulmonary artery reached 78.1% [8]. Pulmonary artery involvement in patients with TA increases the likelihood of misdiagnosis or delayed diagnosis because of the nonspecific respiratory manifestations and lack of symptoms of systemic vessel involvement [9]. PH is a late manifestation of PA that indicates a weaker response to treatment and a poor prognosis [3]; thus, it is vital to achieve an early diagnosis of PA in patients with TA.

This study was performed to analyze the clinical manifestations, imaging features, and prognosis of TA with PA, focusing on the difference between patients with and without PH. Additionally, the early manifestations of PA were explored so that diagnosis can be achieved as early as possible before the occurrence of PH.

## Methods

### Study population

Patients with TA who were hospitalized at Peking Union Medical College Hospital (PUMCH) from January 1986 to December 2015 were identified from the medical records system. Patients with TA who fulfilled the criteria for pulmonary artery involvement were included.

### Inclusion criteria

1. Satisfaction of the diagnostic criteria for TA as defined by the American College of Rheumatology [10].
2. Pulmonary artery involvement as determined by satisfaction of at least one of the following three items: (1) computed tomography pulmonary angiography (CTPA), pulmonary perfusion imaging, or pulmonary arteriography findings suggestive of thickening of the vessel wall or stenosis or occlusion of the pulmonary artery branches; (2) ^18^F-fluorodeoxyglucose positron emission tomography/computed tomography (PET/CT) findings suggestive of high intake of local radioactive material in the pulmonary artery wall; or (3) transthoracic echocardiography findings consistent with presumptive PH without left ventricular disease. PH was defined as an estimated pulmonary artery systolic pressure (PASP) of >50 mmHg and peak tricuspid regurgitation velocity (TRV) of >3.4 m/s, which suggests a high probability of PH according to the European Society of Cardiology/European Respiratory Society PH guideline [11, 12]. Estimation of the PASP was based on the peak TRV, taking into account the right atrial pressure as described by the simplified Bernoulli equation. The right atrial pressure was estimated by echocardiography and was based on the diameter and respiratory variation in diameter of the inferior vena cava [12, 13].

### Characteristics

All patients’ case records and imaging findings were retrospectively reviewed. Observation indices included demographic information, clinical manifestations, radiologic findings, laboratory examination findings, diagnostic procedures, therapeutic interventions, and outcomes. Patients with TA with pulmonary artery involvement were further divided into a PH group and non-PH group for comparison. The prognostic information was collected by telephone follow-up.

### Statistical analysis

Data were analyzed using SPSS 17.0 (SPSS Inc., Chicago, IL, USA) and are expressed as median (range). The measurement data were analyzed by analysis of variance or the Mann–Whitney U test to compare within- or between-group differences. The enumeration data were analyzed by the χ^2^ test. Survival rate analysis was performed using Kaplan–Meier and Cox regression methods. A *P* value of <0.05 was considered statistically significant.

## Results

### General information

**Fig. 1:**
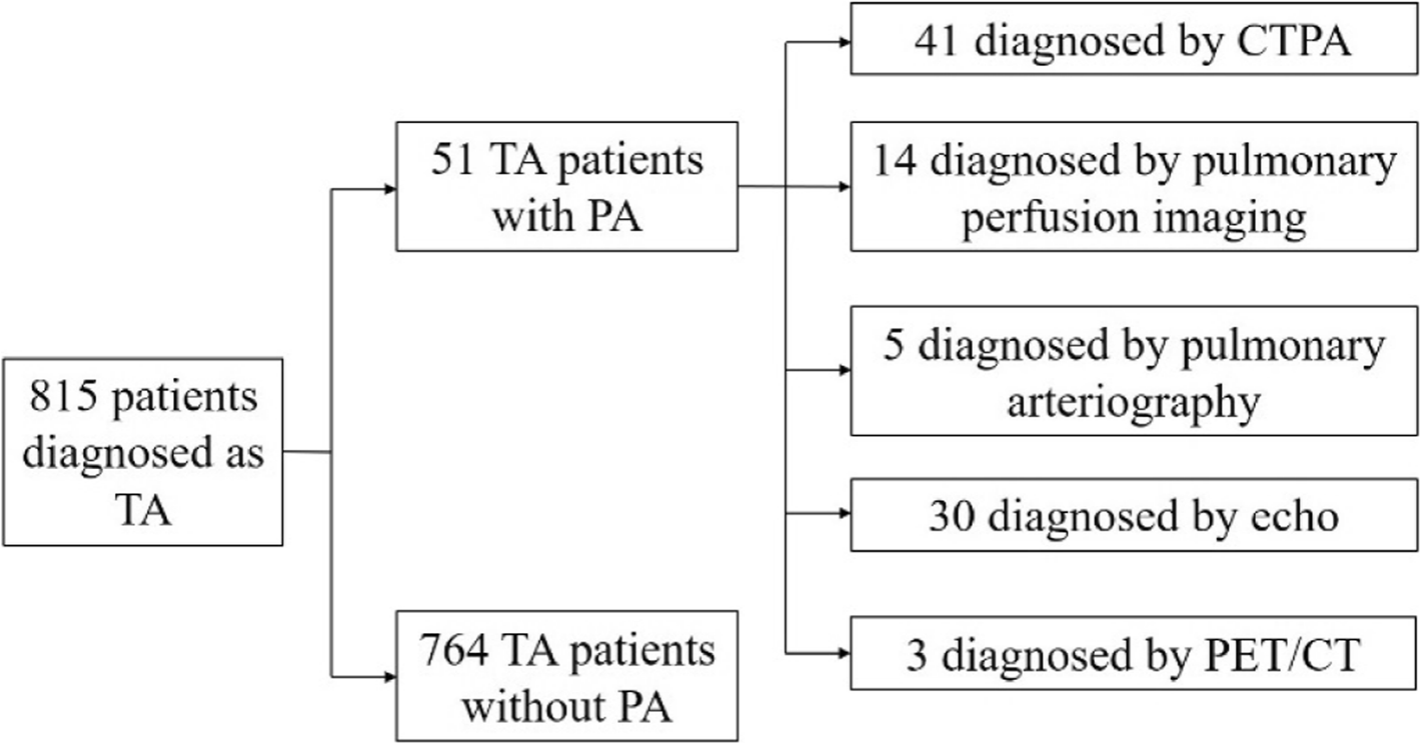
Diagnostic flow chart of patients who had Takayasu’s arteritis with pulmonary artery involvement

**Fig. 2:**
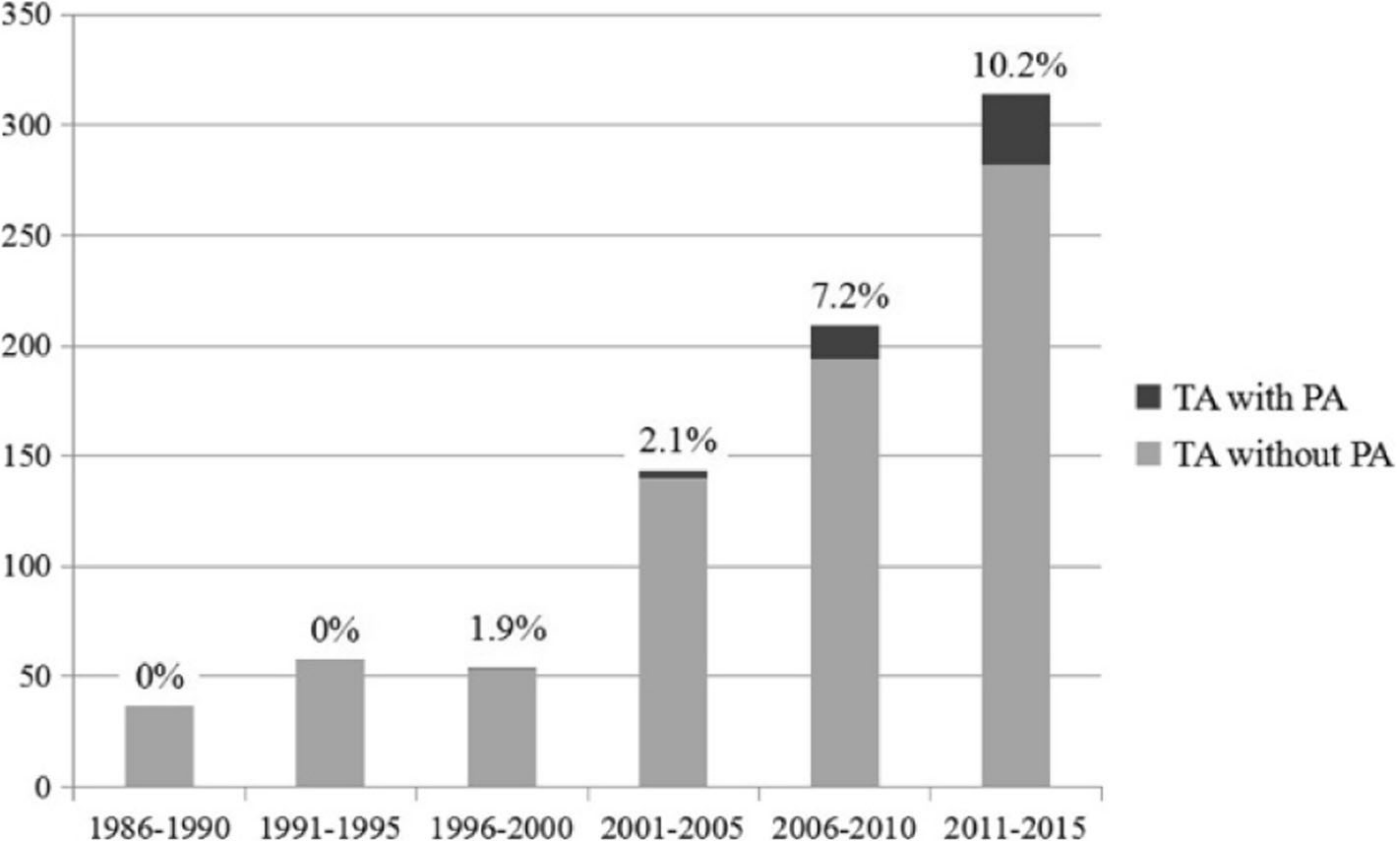
Number of patients with Takayasu’s arteritis (TA) and proportion of patients with pulmonary arteritis (PA) among those with TA gradually increased over 30 years

From January 1986 to December 2015, a total of 815 patients with TA were hospitalized in PUMCH, among whom 51 (6.26%) were found to have PA (Fig. 1). According to their date of diagnosis, the patients were divided into six groups at 5-year intervals. The number of patients with TA increased over time (*n* = 37, 58, 54, 143, 209, and 314). In addition, the proportion of patients with PA among those with TA also showed an upward trend with time (0.0, 0.0, 1.9, 2.1, 7.2, and 10.2%) (Fig. 2).

The 51 patients comprised 39 females and 12 males at a male:female ratio of 1.0:3.2; the median age of the patients was 33 (14–67) years. Among the 51 patients with PA, 30 (58.8%) were diagnosed with presumptive PH [median estimated PASP, 84.5 (52–139) mmHg; median peak TRV, 4.25 (3.5–5.6) m/s] by transthoracic echocardiography. This number of patients with PH accounted for 93% of those in the previous 5 years (2006–2010) but for only 50% of those in the more recent 5 years (2011–2015). The median duration from onset to hospitalization was 28 (1–540) months. The median duration from disease onset to hospitalization was longer in the PH group [45.5 (1–540) months] than in the non-PH group [17 (1–120) months, *P* = 0.009] (Table 1).

**Table 1:** demographics, clinical manifestations, imaging features of Takayasu’s arteritis patients with pulmonary arteritis

	total (*n* = 51)	non-PH (*n* = 21)	PH (*n* = 30)	*P*
Sex (female)	39 (76.5%)	15 (71.4%)	24 (80%)	0.518
Age (year)	33 (14–67)	30 (15–65)	35.5 (22–67)	0.196
Median duration of disease (months)	28 (1–540)	17 (1–120)	45.5 (1–540)	0.009*
Clinical symptoms				
Cough	34 (66.7%)	15 (71.4%)	19 (63.3%)	0.546
Hemoptysis	24 (47.1%)	14 (66.7%)	10 (33.3%)	0.019*
Dyspnea	36 (70.6%)	11 (52.4%)	25 (83.3%)	0.017*
Chest pain	23 (45.1%)	15 (71.4%)	8 (26.7%)	0.002*
Palpitation	12 (23.5%)	4 (19.0%)	8 (26.7%)	0.767
Fever	22 (43.1%)	13 (61.9%)	9 (30.0%)	0.024*
Lab tests				
WBC (×10^9^/L)	9.07 (4.06–18.46)	9.80 (8.92–17.00)	8.48 (4.06–18.46)	0.246
ESR (mm/h)	16 (1–178)	34 (2–178)	8.5 (1–140)	0.027*
CRP (mg/dL)	7.17 (0.11–238.31)	13.7 (0.37–238.31)	5.65 (0.11–155.00)	0.284
Radiological features				
Cavity	7 (13.7%)	5 (23.8%)	2 (6.7%)	0.181
Nodule	25 (49.0%)	11 (52.4%)	14 (46.7%)	0.688
Pleural thickening	27 (52.9%)	10 (47.6%)	17 (56.7%)	0.524
irregular linear opacities	26 (51.0%)	11 (52.4%)	15 (50.0%)	0.867
Patchy opacities	27 (52.9%)	12 (57.1%)	15 (50.0%)	0.615
Subpleural wedge-shaped shadow	13 (25.5%)	10 (42.9%)	3 (10.0%)	0.002*
Mosaic perfusion	3 (5.9%)	0	3 (10.0%)	0.259
Extrapulmonary vascular involvement	39 (76.5%)	13 (61.9%)	26 (86.7%)	0.040*
Aorta	25 (49.0%)	8 (38.1%)	17 (56.7%)	0.192
Carotid artery	32 (62.7%)	10 (47.6%)	22 (73.3%)	0.062
Vertebral artery	6 (11.8%)	3 (14.3%)	3 (10.0%)	0.979
Subclavian artery	30 (58.8%)	10 (47.6%)	20 (66.7%)	0.174
Mesenteric artery	6 (11.8%)	0	6 (20.0%)	0.036*
Renal artery	18 (35.3%)	4 (19.0%)	14 (46.7%)	0.083
Iliac artery	3 (5.9%)	1 (4.8%)	2 (6.7%)	1.000
Coronary artery	2 (3.9%)	1 (4.8%)	1 (3.3%)	1.000

Values in parentheses indicate percentage or range. *CRP* C-reactive protein, *ESR* Erythrocyte sedimentation rate, *WBC* White blood cells. *P < 0.05

### Clinical manifestations

Respiratory symptoms occurred in 48 patients (94.1%): dyspnea, 70.6% (36/51); cough, 66.7% (34/51); hemoptysis, 47.1% (24/51); and chest pain, 45.1% (23/51). Respiratory symptoms were the initial manifestation in 37 patients (72.5%). Patients without PH were more likely to have symptoms such as fever (61.9% vs. 30.0%, *P* = 0.024), chest pain (71.4% vs. 26.7%, *P* = 0.002), or hemoptysis (66.7% vs. 33.3%, *P* = 0.019) but were less likely to feel dyspnea than patients with PH (52.4% vs. 83.3%, *P* = 0.017) (Table 1).

In terms of the distribution of involved vessels, 12 of 51 patients (23.5%) only had PA and no evidence of extrapulmonary large artery involvement. Extrapulmonary vascular involvement occurred in 39 patients, including the aorta in 25 patients, carotid artery in 32, subclavian artery in 30, renal artery in 18, vertebral artery in 6, mesenteric artery in 6, iliac artery in 3, and coronary artery in 2.

### Laboratory examinations

The leukocyte count in peripheral blood was 9.07 (4.06–18.46) × 10^9^/L, and the neutrophil count was 5.72 (3.56–16.29) × 10^9^/L. The erythrocyte sedimentation rate (ESR) was 16 (1–178) mm/h and C-reactive protein level was 7.17 (0.11–238.31) mg/dL. The ESR was markedly higher in the patients without than with PH (34 vs. 8.5 mm/h, *P* = 0.027) (Table 1).

### Imaging findings

**Fig. 3:**
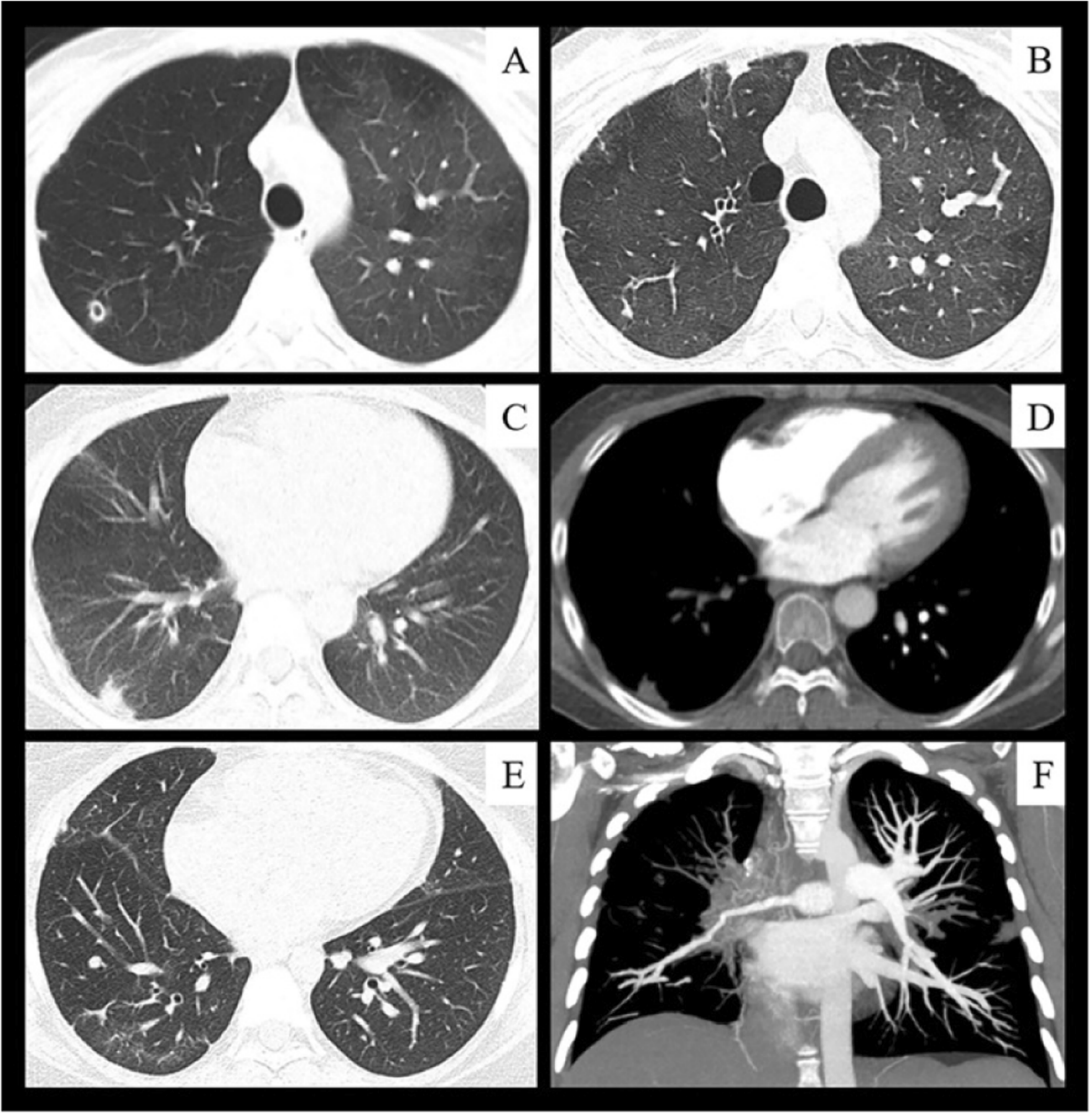
Imaging findings of pulmonary arteritis in patients with Takayasu’s arteritis. **a**, Axial computed tomography (CT) image shows mosaic perfusion with reduced vessels in the darker lung (right lung), indicating occlusive vascular disease; a thin wall cavity is present in the right upper lung, consistent with pulmonary infarction. **b**, CT image obtained 2 years later shows a healing residual lesion from the cavity of the right upper lobe; subpleural scarring is also present. **c**, CT image shows a subpleural wedge-shaped opacity suggestive of pulmonary infarction. **d**, Contrast-enhanced CT image shows corresponding pulmonary artery occlusion in the right lower lobe. **e**, CT image shows peripheral scarring from previous infarcts in the right lower lung. **f**, Coronal reformatted image from CT pulmonary angiography in the same patient as in (**e)** shows occlusion of the right upper lobe artery and stenosis of the right interlobar artery and lower lobe artery

**Fig. 4:**
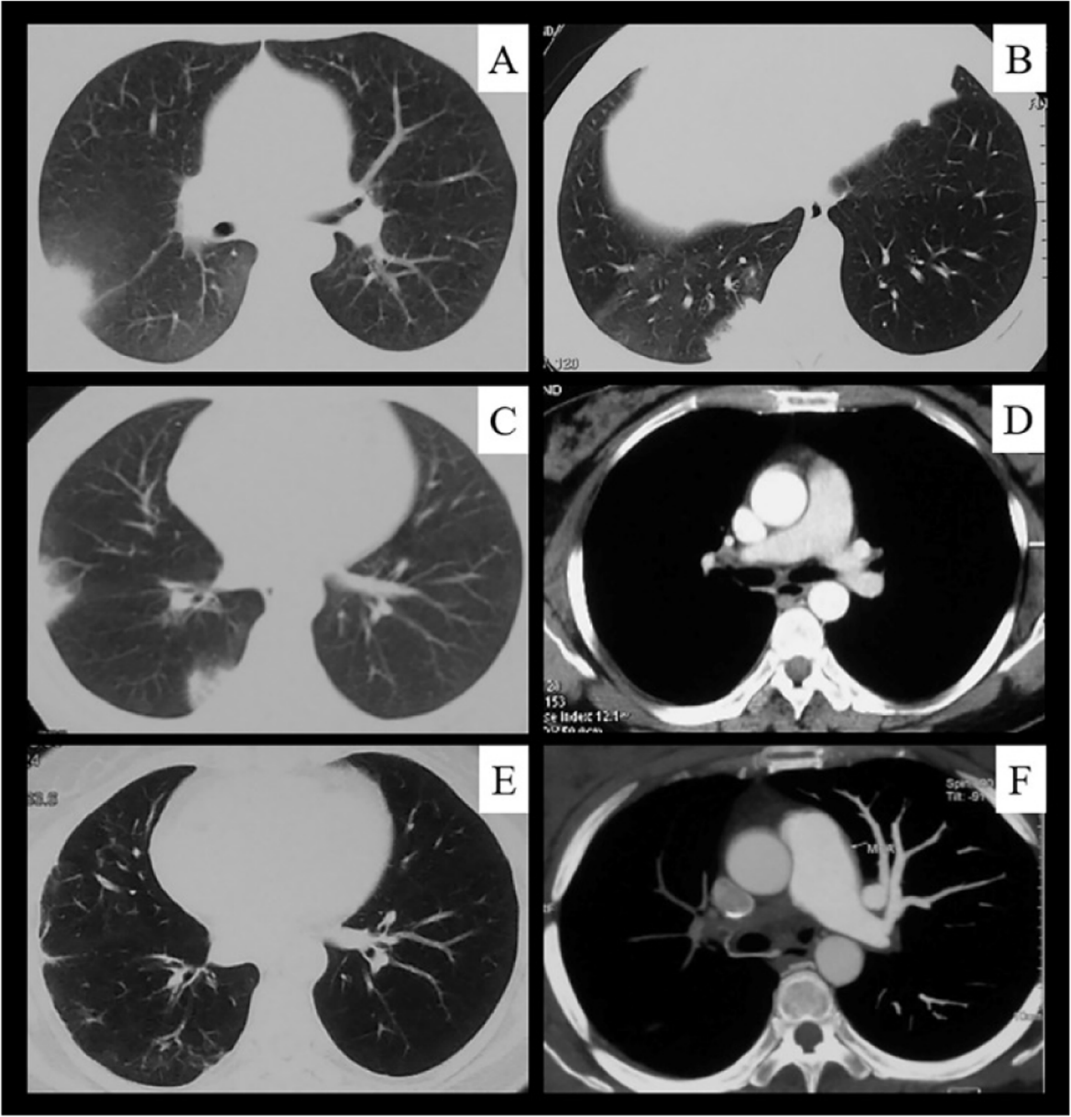
Serial computed tomography (CT) images in a 40-year-old woman with Takayasu’s arteritis. The patient was admitted to our hospital because of shortness of breath. She had developed recurrent chest and back pain and hemoptysis 4 years previously. **a–c**, CT images show recurrent subpleural wedge-shaped opacities during the initial 6 months after disease onset. **d** CT pulmonary angiography (CTPA) image obtained at the same time as in (**c**) showed right pulmonary artery stenosis. **e**, four years later, a CT image shows peripheral scarring from previous infarcts. **f**, CTPA image obtained at the same time showed right pulmonary artery occlusion

Chest CT showed pulmonary parenchymal involvement in 42 of 51 (82.4%) patients with TA involving the pulmonary artery: pleural thickening in 27 patients (52.9%), patchy opacities in 27 (52.9%), irregular linear opacities in 26 (51.0%) (Fig. 3b, e and Fig. 4e), nodules in 25 (49.0%) (Fig. 3a), subpleural wedge-shaped shadows in 13 (25.5%) (Fig. 3c and Fig. 4a to c), cavities in 7 (13.7%) (Fig. 3a), and mosaic perfusion in 3 (5.9%) (Fig. 3a). The proportion of subpleural wedge-shaped shadows was significantly higher in the non-PH group than PH group (χ^2^ = 9.204, *P* = 0.002) (Table 1).

Of the 44 patients who underwent pulmonary vascular imaging, 41 underwent CTPA showing pulmonary artery stenosis in 33 patients (Fig. 3f and Fig. 4d), pulmonary artery occlusion in 32 (Fig. 3d, f and Fig. 4f), pulmonary artery wall thickening in 14, and pulmonary artery expansion in 7. Five patients underwent pulmonary arteriography, which showed pulmonary artery stenosis and occlusion. Pulmonary perfusion imaging in 14 patient revealed perfusion defects.

### Diagnosis and differential diagnosis

Forty-four patients with PA were diagnosed by pulmonary vascular imaging examinations (CTPA in 41, pulmonary perfusion imaging in 14, and pulmonary arteriography in 5; at least two pulmonary vascular imaging abnormalities were found in 14 patients). Three patients underwent systemic PET/CT scans showing pulmonary artery involvement. Six patients were diagnosed only by echocardiography (Fig. 1).

Forty-two (82.4%) patients had been diagnosed with other diseases before the diagnosis of PA, including pulmonary infection in 21 (50.0%), pulmonary tuberculosis in 13 (31.0%), idiopathic PH in 9 (21.4%), pulmonary embolism in 5 (11.9%), bronchial dilatation in 4 (9.5%), myocarditis in 2 (4.8%), heart failure in 2 (4.8%), lung malignancies in 1 (2.4%), and allergic pneumonia in 1 (2.4%).

### Treatment and prognosis

Fifty-one patients were treated with a glucocorticoid at a starting dose of 0.5 to 1.0 mg/kg/day; 45 patients received immunosuppressive therapy (39 with cyclophosphamide, 4 with methotrexate, 3 with azathioprine, 1 with tacrolimus, 1 with cyclosporine A, 1 with mycophenolate mofetil, and 1 with triptolide; 3 patients received treatment with two or more disease-modifying antirheumatic drugs). Seventeen patients received aspirin antiplatelet therapy, 17 were treated with warfarin anticoagulation, and 5 were treated with both aspirin and warfarin.

**Fig. 5:**
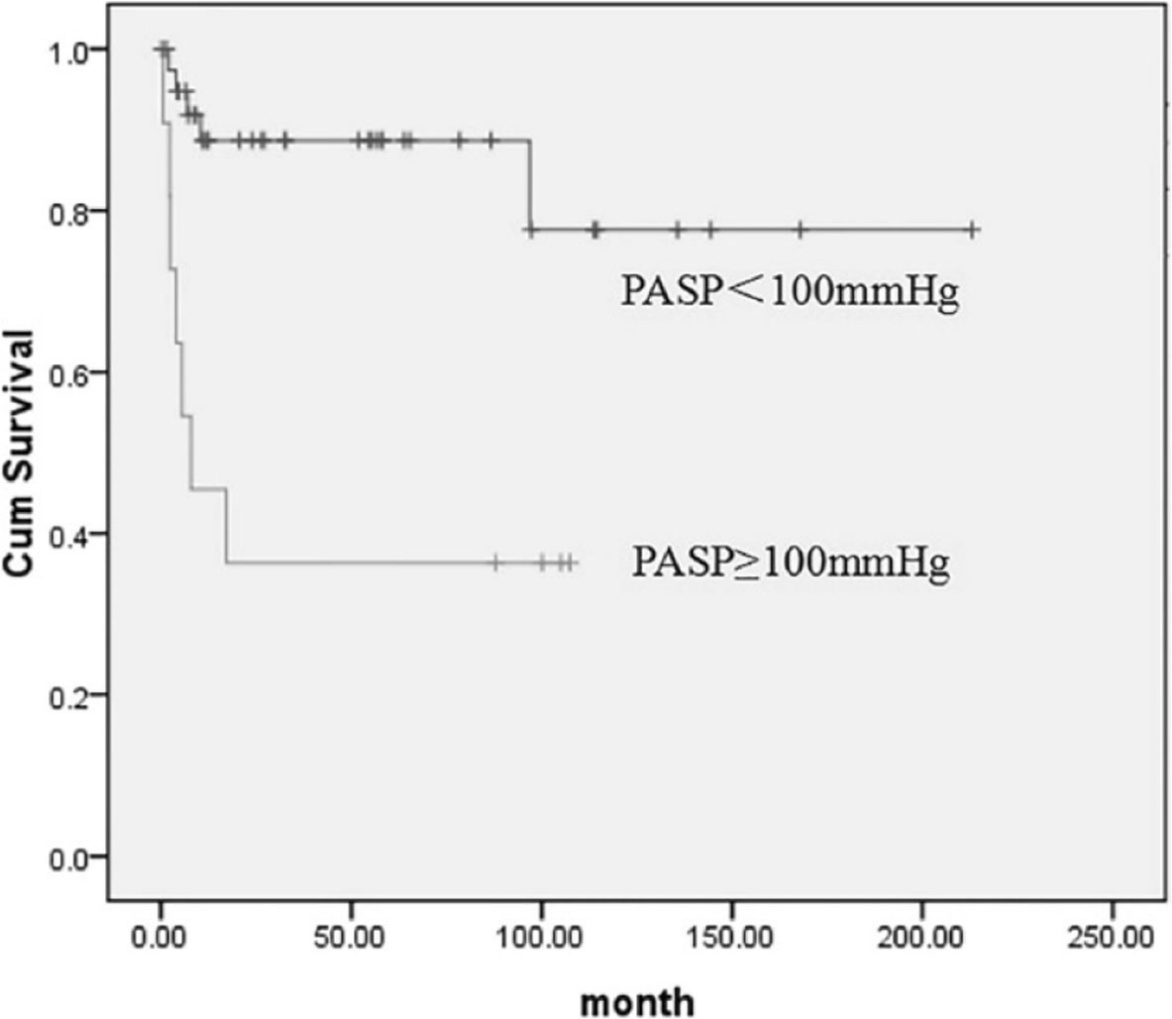
Kaplan–Meier analysis of the risk of death or repeated hospital admissions. Patients with a PASP of ≥100 mmHg had a greater risk of death or repeated hospital admissions than patients with a PASP of <100 mmHg

Fifty-one patients with TA involving the pulmonary artery were followed up for 48(1–212) months. Three of these patients died, and all were in the PH group. Eleven patients were readmitted to the hospital because of TA-related clinical manifestations, including nine patients in the PH group and two in the non-PH group. Patients without PH had significantly lower mortality and readmission rates than those with PH (9.5% vs. 33.3%). The Kaplan–Meier survival analysis showed that the risk of death or repeated hospital admissions significantly decreased if the PASP was <100 mmHg (Fig. 5). Cox regression analysis showed that the risk of death or readmission significantly increased if the PASP was ≥100 mmHg in patients with PA after adjusting for the patients’ demographics (age and sex), PASP, ESR, CRP, and presence or absence of extrapulmonary involvement [hazard ratio (HR), 18.00; 95% confidence interval, 3.51–92.29; *P* = 0.001]. An ESR of ≥20 mm/h was the key factor for reducing the risk of death or repeated hospital admissions (HR, 0.15; 95% confidence interval, 0.03–0.78; *P* = 0.024).

## Discussion

In the present study, we reviewed 815 patients with TA during a 30-year period in a single center and found that (1) PA was present in 6.3% of patients with TA and that this proportion increased over time, (2) 58.8% (30/51) of patients with PA developed PH, and (3) 82.4% (42/51) of patients with PA had lung parenchymal lesions on CT scans, representing indirect signs of pulmonary artery involvement. Compared with the PH group, the non-PH group had different clinical features and imaging findings and a better prognosis.

The proportion of PA among patients with TA is still unclear and varies greatly among previous studies, which may be related to differences between study populations or diagnostic methods [5, 6, 14–18]. In the present study, we found that PA occurred in 6.3% of patients with TA. Notably, the rate of PA in patients with TA continually increased over time and reached 10% of patients with TA. The increasing diagnosis rate was due to the increasing awareness of TA and pulmonary vasculitis among clinicians in our center; it was also associated with improvements in diagnostic methods. For example, CTPA and PET/CT have become more widely used during the past 10 years in our clinic. This result also suggests that a previously considerable number of patients who had TA with PA were probably underdiagnosed, implying that more attention should be paid to screening these patients in future.

The symptoms of PA are insidious and nonspecific. The main symptoms in this study were dyspnea (70.6%), cough (66.7%), hemoptysis (47.1%), and chest pain (45.1%), while 5.9% of patients had no respiratory symptoms. This finding is consistent with a meta-analysis by Toledano et al [3]. Patients who have TA with pulmonary artery involvement have occult-onset and nonspecific symptoms, making early diagnosis difficult. Previous studies have shown that the delays in diagnosis and treatment range from 3 to 72 months in patients with PA [9, 19, 20]. In the present study, the median time from the initial symptoms to definitive diagnosis was 13.5 (1–186) months. In addition, 80% of patients in this study were diagnosed with other diseases at their first visit, mostly pulmonary infection, pulmonary tuberculosis, idiopathic PH, and pulmonary embolism, consistent with previous studies [9, 20, 21]. Thus, identification of PA in patients with TA is challenging for physicians.

Accurate diagnosis of pulmonary artery involvement virtually always depends on imaging studies because the clinical manifestations and laboratory test results are usually nonspecific. Typical CT manifestations of pulmonary artery involvement include artery wall thickening and enhancement in the early disease stages and luminal stenosis or occlusion in the chronic stages [22, 23]. Few previous studies or monographs have mentioned changes in the lung parenchyma [22–24]. Occasional case reports have shown that pulmonary infarction is a rare clinical manifestation in patients with TA [25, 26] and that chest CT commonly shows a subpleural wedge-shaped shadow [27, 28]. Unlike the results of previous studies, our results showed that up to 82.4% of patients who had TA with PA showed lung parenchymal changes on chest CT, which might have been secondary to pulmonary vasculitis. The subpleural wedge-shaped shadow was probably the pulmonary infarction caused by PA inflammation-induced vascular stenosis, occlusion, or in situ thrombosis. Pulmonary cavities were probably due to pulmonary parenchymal ischemia and necrosis. Mosaic perfusion indicated vascular occlusion and reduced blood flow. Peripheral irregular linear opacities indicated prior lung infarction. Our results showed that in addition to direct signs such as pulmonary artery wall thickening or narrowing, the imaging findings of pulmonary vasculitis also have indirect signs; i.e., changes in the lung parenchyma that indicate pulmonary artery involvement and should prompt further pulmonary vascular investigations such as CTPA.

TA-related PH is classified as group 4 PH (i.e., chronic thromboembolic PH and other pulmonary obstruction) [12]. The present study showed that PH occurred in 58.8% of patients with PA, consistent with previous studies (42.2–78.1%) [3, 8]. All three deaths in this study were related to severe PH, suggesting that PH might be related to a poor prognosis and increased mortality. Therefore, early diagnosis is particularly important. Notably, the rate of PH decreased during the most recent 5 years (2011–2015) compared with the previous 5 years (2006–2010) in our center (50.0% vs. 94.3%, respectively). This result prompts us to consider the possibility of further reducing the incidence of PH in patients with TA with pulmonary artery involvement in the future. The present study showed that the clinical and imaging findings differed between the PH and non-PH group. Compared with the PH group, the non-PH group was characterized by a shorter disease course; more symptoms such as fever, chest pain, and hemoptysis; an increased ESR; and a higher incidence of subpleural wedge-shaped shadows on chest CT, suggesting that patients in the non-PH group were in the early and active inflammatory stage. Therefore, the early manifestations of PA include recurrent fever, chest pain, hemoptysis, an elevated ESR, and recurrent subpleural wedge-shaped shadows, which should raise suspicious for PA and prompt further vascular imaging investigations. This may allow TA to be diagnosed before the occurrence of PH.

Our study has several limitations. It was retrospective in nature, not all patients routinely received pulmonary vascular-related examinations, and the diagnostic approaches were inconsistent. Right ventricular catheterization, which is the gold standard for the diagnosis of PH, was not performed. Thus, the exact incidence of PH in patients with TA is unknown and should be confirmed in future studies. Additionally, we only included hospitalized patients with TA, whose condition was more severe than that of outpatients, possibly leading to selection bias. Therefore, a multicenter prospective cohort study will be required to confirm our results.

## Conclusions

The clinical manifestations of TA involving the pulmonary artery are nonspecific, making this condition subject to misdiagnosis and delayed diagnosis. PH often complicates PA and is associated with a poor prognosis. Early clinical manifestations such as repeated fever, chest pain, hemoptysis with or without dyspnea, and recurrence of subpleural wedge-shaped shadows on chest CT should arouse suspicion for PA in patients with TA and prompt further vascular imaging investigations. This may allow TA to be diagnosed early, before the occurrence of irreversible stenotic and fibrotic vascular lesions.

## Data Availability

The datasets generated and analysed during the current study are available in the *ClinicalTrials* repository [https://clinicaltrials.gov/ct2/results?recrs=&cond=&term=NCT03189602&cntry=&state=&city=&dist=].

## Abbreviations

CRP: C-reactive protein
CTPA: Computed tomography pulmonary angiography
ESR: Erythrocyte sedimentation rate
PA: Pulmonary arteritis
PASP: Pulmonary arterial systolic pressure
PET/CT: Positron emission tomography/computed tomography
PH: Pulmonary hypertension
PUMCH: Peking Union Medical College Hospital
TA: Takayasu’s arteritis
TRV: Tricuspid regurgitation velocity
WBC: White blood cells
HR: Hazard ratio
